# Partnership Preferences in Modern Migration Societies: Religious Homophily Among Young Muslims and Christians in Germany

**DOI:** 10.1007/s10680-024-09728-0

**Published:** 2025-01-16

**Authors:** Stefanie Heyne, Jana Kuhlemann, Irena Kogan

**Affiliations:** Mannheim Centre for European Social Research (MZES), University of Mannheim, Mannheim, Germany

**Keywords:** Partner choice, Factorial survey experiment, Homophily, Religion, Gender differences

## Abstract

Preferences for homogamous partnerships, not only in terms of having a partner with the same ethnicity, but one with the same religion, are an important factor in explaining low levels of interethnic partnerships in Western countries. However, previous research has rarely explicitly focused on the role of preferences for partnership formation patterns. Using data from a factorial survey experiment, which was implemented in the 9th wave of the “Children of Immigrants Longitudinal Survey in Four European Countries” among young adults in Germany (CILS4EU-DE), this study explores patterns of religious homophily in partnership preferences among young adults in Germany. It thereby specifically focuses on individuals belonging to two major religious groups: Christians and Muslims. It investigates the effects of religious denomination, religiosity, type of intimate partnership, and gender differences for religious homophily in partnership preferences. Both male and female respondents were found to prefer partners of the same religious denomination, with this effect being stronger among Muslims than Christians. Muslim respondents showed no or little disapproval of more strongly religious partners, whereas Christian respondents showed strong disapproval of more religious partners. Among Christian and male Muslim but not among female Muslim respondents, preferences for religious homophily were lower for casual partnerships than for marriage. Overall, the findings suggest that religious homophily and religion play an important part in shaping partnership choices among young Christian and Muslim adults in Germany.

**Supplementary Information:**

The online version contains supplementary material available at 10.1007/s10680-024-09728-0.

## Introduction

In recent decades, the ethnic composition of the populations of many Western societies has changed enormously towards higher proportions of immigrants and their descendants.^1^ Still, rates of intermarriage and other interethnic romantic partnerships remain rather low (Adsera & Ferrer, 2014), with immigrants as well as their descendants being more likely to marry co-ethnics than members of other ethnic groups or members of the majority population (Huschek et al., 2012; Muttarak, 2010). Even though intermarriage, defined as the marital union between members of two different ethnic, cultural, or religious groups, has been on the rise, it is still far less common than one would expect (Qian & Lichter, 2011; Rosenfeld, 2008). Previous research has described different reasons for this high share of ethnically homogeneous partnerships and has emphasised that not only the availability of potential partners, but also individual preferences and third-party influences are important in this context (for an overview see Kalmijn, 1998).

In this article we focus on preferences for homogamy in terms of religion as an important source of social norms (Mahoney et al., 2003; McQuillan, 2004). Despite the well-advanced process of secularisation in many Western European countries and a diminishing role of religion for the members of the majority population, religion remains as important as ever in the context of migration, as immigrants have brought not only linguistic and cultural diversity, but also religious pluralism to the receiving societies (Voas & Fleischmann, 2012).

Against this background, this paper analyses the importance of religion for partnership preferences of young adults in Germany. The persistent inflow of immigrants into Germany over past few decades has made the German society highly diverse, with more than one quarter of the population being either immigrants themselves, descendant of immigrants, or born to at least one parent who did not have German citizenship at birth (Federal Statistical Office, 2022). This development is also reflected in greater religious plurality. Germany was originally shaped by Christianity, although increasing secularisation and the socialist past in East Germany have led to a substantial proportion of people who do not belong to any religion (Pollack & Pickel, 2007), with a 2018 survey showing that 26.9% of respondents in Germany indicated no religious affiliation, with 16.6% in West Germany and 68.3% in East Germany (Bundeszentrale für politische Bildung, 2020). Furthermore, the immigration of “guest workers” from Turkey in the 1950s–1970 s, followed by family reunification, and a subsequent influx of refugees—particularly in recent years—have led to a sharp increase in the number of people of Muslim faith in Germany, estimated at more than 5 million by 2020 (Pfündel et al., 2021).

Previous research for Germany has shown low levels of marriage and partnerships between immigrants, their offspring, and members of the majority population (González-Ferrer, 2006; Kalter & Schroedter, 2010). Intermarriage rates are especially low among immigrants and their descendants with high levels of religious identification (Carol et al., 2014). The importance of religion has also been shown in previous studies in several Western countries where disapproval of interethnic, interracial, or interfaith unions was particularly strong among Muslims (Carol & Teney, 2015), especially if they were highly religious (Buunk & Dijkstra, 2017; Cila & Lalonde, 2014).

Using a factorial survey experiment (FSE) implemented in the German part of the “Children of Immigrants Longitudinal Survey in Four European Countries” (CILS4EU-DE) data, we extend the existing research in three regards. First, the CILS4EU-DE data make it possible to analyse partnership preferences in terms of religion among young adults belonging to the Christian and the Muslim faith, with the latter group being projected to comprise an increasingly larger proportion of the population of many European countries in the future. A FSE also enables a more precise measurement of preferences for religious homogamy compared to direct questions on partnership preferences utilised in previous studies. This is because the FSE allows for the disentanglement of highly correlated characteristics of potential partners, such as religious denomination and level of religiosity.

Second, we consider that the receptiveness to interethnic partnerships may vary based on the nature of romantic relationships, as prior research has indicated variations in the degree of (ethnic) homogamy depending on the type of partnership (Blackwell & Lichter, 2004). Single studies have found similar patterns regarding preferences towards interethnic or interracial partnerships (for Muslim young adults in Canada, see Cila and Lalonde (2014), for second-generation immigrant young adults with Moroccan and Turkish background in the Netherlands, see Buunk and Dijkstra (2017), and for white US-Americans, see Herman and Campbell (2012)).

Third, we explore gender differences in preferences for religious homogamy, considering that intermarriage rates vary by both ethnic group and gender (Choi & Tienda, 2017; Hannemann et al., 2018). Research has generally found lower intermarriage rates among Muslim women compared to Muslim men (Carol, 2016; González-Ferrer, 2006). This has been attributed to factors such as lower acceptance of intergroup romantic relationships for women by third parties, such as parents (Hartung et al., 2011; Munniksma et al., 2012; Van Niekerk & Verkuyten, 2018) and gendered preferences for intergroup partners (Carol & Teney, 2015; Feliciano et al., 2009; Herman & Campbell, 2012).

In the present study, we analyse the partnership preferences of young adults in Germany along religious lines using a FSE implemented in the CILS4EU-DE panel survey, in which young adults reported their willingness to engage in different types of romantic partnerships in several vignettes that comprised the description of a fictitious partner. These potential partners were described in terms of their religious denomination, being either Christian or Muslim, and in their level of religiosity, indicating whether religion played an important role or no big role in their lives. In the vignettes, we additionally varied the type of the romantic partnership, differentiating between casual partnerships, committed partnerships, and marriages. Using information on the respondents’ own religious denomination and gender, we tested (1) whether young Christian and Muslim adults in Germany prefer religiously homophile partnerships, (2) whether this preference differs by the level of commitment in these partnerships, and (3) whether religious homophily is more important for women than for men.

## Theoretical Background and Hypotheses

### The Role of Preferences in Partner Choice

Individual preferences have been described as one of the major factors in the explanation of the formation of homogamous partnerships (Kalmijn, 1998; McPherson et al., 2001). According to the theory of new home economics (Becker, 1973) and the status exchange theory (Merton, 1941), romantic partnerships are ultimately unions formed in order to accumulate resources that can be used to produce desired commodities. This accumulation of resources can be maximised through the division of labour and the exchange of different types of resources between partners. However, concerning cultural resources and aspects related to individuals’ identity, research suggests that individuals tend to seek similarity (Becker, 1973; Kalmijn, 1998), as it tends to reduce conflict within partnerships (McPherson et al., 2001) and facilitates the attainment of various commodities, including emotional well-being or marital harmony (Clarkwest, 2007). This is in line with social exchange theory (Thibaut & Kelley, 1978), which states that similarity in certain traits of partners is favourable because it produces partnership stability, and with the cultural matching framework, which suggests that individuals prefer partners with shared values and similar cultural resources (Schwartz, 2013).

Individual preferences are, however, not independent from other factors contributing to the explanation of homogamous partnerships, such as third-party preferences and the opportunity to meet potential partners. Individual preferences are influenced by the preferences of third parties such as parents via socialisation (Carol, 2014; Huijnk & Liefbroer, 2012; Weißmann & Maddox, 2016), and Carol (2014) even reports that stronger parental homogamy preferences are associated with offspring’s lower outgroup contacts via offspring’s own preferences. Previous research has also emphasised the importance of third parties’ direct impact on individual preferences via social pressure (Carol et al., 2014; Kalmijn, 1998; Weißmann & Maddox, 2016). It is important to note that partnership preferences are also likely to change over the life course and be influenced by experiences of partnerships earlier in life since romantic partnerships during adolescence can be seen as a practice range for more serious future partnerships (Bouchey & Furman, 2003).

### The Role of Religion for Partnership Preferences

Although religion has been found to be losing importance for partnership formation in the context of modernisation and increasing secularisation in Western European countries (e.g. Kalmijn, 1991), continuous migration and the accompanying religious pluralisation of the population have made the role of religion for partnership formation salient again. Accordingly, religion has been described as one important fault line determining partnership preferences in modern migration societies (Röder, 2015; Van Den Akker et al., 2013).

From a theoretical perspective, religion plays an important role for partnership formation since it provides moral rules and set social norms relevant in the context of partnerships, for instance concerning family values, gender roles, and sexuality (Mahoney et al., 2003; McQuillan, 2004). As described above, shared values and interests are important determinants of partnership preferences from an individual’s perspective. Furthermore, the influence of third parties is particularly relevant in the realm of religion given that most religious denominations contain rules on interfaith partnerships, which are—to a varying degree—enforced by their members and religious institutions. Previous research generally posits that, in comparison to other religions prevalent in Western societies, Islam is characterised by stricter rules and more rigorous enforcement mechanisms (Kalmijn & Kraaykamp, 2018; Kogan & Weißmann, 2020; Röder, 2015).

Previous research on interethnic partnerships in Western countries has described an important boundary between members of Christianity and Islam, which can be attributed to both differences in values as well as variations in the influence of third parties. Although Christianity and Islam share similar values regarding family and sexuality in their origins, they differ in the emphasis placed on these values in their contemporary interpretations. Christianity in (Western) Europe has experienced significant transformations in the context of modernisation, leading to shifts in cultural norms among members of Christian denominations. These changes have further contributed to a diminished influence of religious institutions on both the state and the family. Contrary to that, most countries of the Islamic world are characterised by a lower level of secularisation and a strong influence of religious authorities on the state and the family (Moghadam, 2003; Norris & Inglehart, 2011; Tibi, 1980).

Accordingly, internationally comparative research has shown that Muslims and individuals residing in predominantly Muslim countries tend to endorse more traditional gender role attitudes (e.g. Alexander & Welzel, 2011; Inglehart & Norris, 2003), exhibit lower acceptance of homosexuality (e.g. Adamczyk & Pitt, 2009) and report fewer instance of premarital and extramarital sex (Adamczyk & Hayes, 2012). Differences in family values between Islam and European Christianity are also reflected in demographic behaviour. While Western countries experienced a pluralisation of family forms (Billari & Liefbroer, 2010), marriage and childbirth are still universal in most predominantly Muslim countries (Nauck & Klaus, 2005; Rashad et al., 2005). The divergence in societal attitudes and demographic behaviour between Muslims and non-Muslims is also evident in research on immigrants in Western Europe (Drouhot & Nee, 2019), with studies showing more traditional attitudes towards sexual liberalisation (e.g. Kogan & Weißmann, 2020; Soehl, 2017) and gender equality (e.g. Diehl et al., 2009; Kretschmer, 2018) among Muslim immigrants and their offspring in Germany. Similarly, research has shown that Muslim immigrants have replicated the demographic behaviour and gendered family patterns prevalent in their home countries in Western Europe. Nevertheless, there are observable processes of adaptation to the autochthonous population in Western Europe among the descendants of Muslim immigrants (e.g. Huschek et al., 2011; Milewski, 2011). Given the divide in social norms and values that are important for partnership formation, we overall expect that:

#### H1a

 Preferences for partners belonging to the aligning denomination are higher than preferences for partners belonging to a different religious denomination.

As discussed above, individual preferences are not independent from the role of third parties in partnership formation. Several predominantly Muslim countries are still dominated by traditional patriarchy, in which the family and the authority of a male relative play an important role in all areas of life. In several countries, family law is regulated by Sharia, which sets clear rules for marriage, sexuality, and gender rights, often discriminating against women in areas such as inheritance, parental authority, and the right to divorce. This differs greatly from secular family law in Western countries (Esposito, 2001; Moghadam, 2003). Although this secular family law also applies to Muslims living in Western countries, previous research has suggested that the influence of religious institutions and family remains stronger among Muslim immigrants compared to members of the Christian or non-religious majority (Kogan & Weißmann, 2020). Given a stronger impact of third parties and social control for Muslim compared to Christians, we expect:

#### H1b

 Preferences for partners belonging to the aligning denomination are higher for Muslims than for Christians.

Furthermore, previous research has emphasised that the religious divide between Muslims and Christians in Western societies is partly due to higher levels of religiosity among Muslims than among Christians (Drouhot & Nee, 2019; Foner & Alba, 2008). Given that previous studies found a higher importance of religion among Muslims, particularly in the context of partnership formation (Carol & Teney, 2015), we expect differences in openness to partnerships with religious individuals based on one’s own denomination, so that:

#### H2a

 Muslims show a higher preference for religious partners over non-religious partners, while Christians show a higher preference for non-religious partners over religious partners.

However, given that denominations differ in their actual interpretations of family values, as explained above, the openness towards religious partners should also vary based on the denomination of the potential partner. Accordingly, we expect that:

#### H2b

 Respondents show a higher preference for religious partners belonging to the aligning denomination than for religious partners belonging to a different denomination.

### Religious Homophily by the Level of Partnership’s Commitment

However, it can be assumed that the importance of homophily may vary based on the commitment level within a partnership, a notion commonly referred to as winnowing thesis. It states that partnerships with lower levels of commitment, such as dating or casual partnerships, are less likely to be homogamous with respect to ascribed and achieved traits, compared to partnerships with high levels of commitment, such as marriage (Blackwell & Lichter, 2004). A more preference-focused approach states that this phenomenon is driven by different motivations for partnerships with different levels of commitment. In low-commitment partnerships, individuals may be inclined to explore their options, diminishing the necessity of finding a partner suitable for a long-term partnership. Conversely, in high-commitment partnerships, individuals have higher incentives to find a compatible partner with whom to potentially raise children and spend a long time together (Blackwell & Lichter, 2004). This aligns with Becker’s (1973) new home economics, suggesting that the utility of homogamy increases with the level of commitment within the partnership. As the stakes and incentives to prevent union dissolution increase, there is a heightened value placed on finding a partner who shares similar characteristics. For instance, raising children successfully as a form of utility of a high-commitment partnership is more easily achieved in homogamous partnerships, due to lower conflict and higher probability of the couple staying together. The expectations concerning the utility of a partnership should in turn affect the partnership preferences. Given that individual preferences for religiously homophilic partnerships are rooted in the shared values of both partners, it follows that religious homophily would generally hold greater significance in partnerships characterised by higher levels of commitment. Accordingly, we expect that:

#### H3a

 Respondents show a higher preference for a partnership with a person belonging to the aligning denomination if the partnership is a marriage compared to a less committed partnership.

However, the set of options regarding partnership types that are viable for individuals belonging to a particular religious denomination might be additionally restricted by religious norms, guarded by the religious community and the family. Both Islam and Christianity reject pre- or extramarital sexual relations, although Christian churches are less able to enforce such norms in European countries nowadays due to increased secularisation and liberalisation (Kogan & Weißmann, 2020; Vignoli & Salvini, 2014). Accordingly, partnerships with lower commitment such as causal partnerships, might typically be deemed unsuitable by Muslims, irrespective of their partner’s religious denomination. Nevertheless, given that third-party influence should be lower for low-commitment partnerships, since such unions are easier to keep away from parental or peer scrutiny, such relationships might offer Muslims an opportunity to transcend cultural boundaries (Buunk & Dijkstra, 2017; Wang et al., 2006). In contrast, concealing a marriage from third parties, including parents, peers, and the religious community, represents a more drastic and intricate decision with far-reaching consequences. As such, it is less likely to occur compared to hiding a more casual and low-commitment romantic partnership (Buunk & Dijkstra, 2017). Given that third-party influences are stronger for Muslims than Christians, we expect that:

#### H3b

 The disparity in preferences for partnerships with individuals belonging to the aligning denomination varies more significantly by partnership type among Muslims than among Christians.

### Gender Differences in Religious Homophily

Furthermore, we expect preferences for religious homogamy to be gendered, given the traditional role of women as transmitters of cultural norms and values. This is likely to result in a stronger influence of parents in the transmission of norms of endogamy and greater parental control in the partnership formation process for women compared to men (Carol & Teney, 2015). This gendered influence on partnership formation is likely to be more pronounced in Muslim contexts relative to Christian ones. As described above, Islam is influences by traditional patriarchy values, which create different expectations for the behaviour of male and female family members. While several discriminatory practices of Islamic law do not apply to Muslim immigrants in Western countries, some traditions remain unaddressed by the civil family law in countries like Germany. This is particularly evident in the social norm that family honour depends on adherence of female family members to prescribed gender roles, such as practicing sexual abstinence before marriage. Furthermore, the Quran prohibits Muslim women to marry a non-Muslims partner, whereas this is accepted for Muslim men (Esposito, 2001). As a result, more negative attitudes towards interfaith marriages of daughters than of sons can be observed among Muslims (Van Niekerk & Verkuyten, 2018). Overall, this suggests that Muslim women are more strongly socialised to adhere to partnership norms and are more susceptible to pressure from third parties compared to Muslim men.

Contrary to this, norms concerning partnerships and sexual behaviours are much less gendered in the modern Western European societies. This implies that young Christian men and women face more similar pressures and are subject to more uniform rules of partnership formation compared to Muslim immigrants residing in those same countries (Maddox, 2019). Accordingly, we propose that:

#### H4

 The associations described in hypotheses H1a-H3b are more pronounced for Muslim women than for Muslim men but are not more pronounced for Christian women than for Christian men.

## Data and Methods

For our analyses, we employ a factorial survey experiment (FSE) that was implemented in the 9th wave of the German panel study “Children of Immigrants Longitudinal Survey in Four European Countries” (CILS4EU-DE (Kalter et al., 2024)). The panel started in 2010 as a survey of ninth graders aged 14–15 in Germany, Sweden, the Netherlands, and England. A first-wave sample was obtained through a three-stage sampling methodology. This process involved selecting general schools enrolling ninth graders as the first stage, followed by classes within these schools as the second stage, and finally, all adolescents in these classes as the third stage. To ensure an adequate representation of adolescents with a migration background,^2^ schools with a high proportion of immigrant pupils were intentionally oversampled. After three initial waves, the panel continued in Germany with so far additional six waves (Kalter et al., 2019). For the sixth wave, conducted in 2016, a refreshment sample was added to account for panel attrition across previous waves. This sample was drawn from the same birth cohorts as the original sample, using a municipality-based sampling approach. Sampling for the refreshment sample in wave six relied on name lists from randomly selected municipalities. Individuals on these lists were categorised using name-based procedures to identify those with a potential migration background. Respondents from the refreshment sample completed a life history calendar that recorded their educational, labour market, and partnership trajectories, ensuring that the panel included similar information on these respondents as on those from the original panel sample. The refreshment sample included 3513 respondents in wave six (CILS4EU-DE, 2024).

In wave nine, which was collected in 2022, respondents were around 26–28 years old. The overall participation rate in this wave was 87.1% (from a gross sample of 4554 young adults), resulting in a total of 4196 interviews conducted across three modes: web, postal, and telephone. Of these respondents, 1957 were part of the initial panel first surveyed in the year 2010. The other 2239 respondents were part of the refreshment sample drawn in 2016 (for details, see Soiné et al. (2024)). The FSE was implemented in the postal (PAPI) and the web-based (CAWI) mode of the data collection of wave nine, with a non-response rate of 0.5% for the vignette module among the 3812 respondents that have participated in the CAWI and PAPI mode (for details, see Heyne et al. (2024)).

The factorial survey method combines features of traditional surveys with experimental designs, allowing for the manipulation of multiple variables simultaneously. Respondents of a survey are presented with different hypothetical scenarios, known as vignettes, of situations that they are asked to rate on a scale. These vignettes contain multiple dimensions that are experimentally varied, allowing for the manipulation of multiple variables simultaneously. The goal is to assess the relative importance of each dimension. In the employed FSE, each respondent received four different vignettes that comprised the description of a potential partner. Respondents were then asked to evaluate their willingness to engage in a romantic partnership with the described partner on a 11-point scale in the CAWI mode and a 10-point scale in the PAPI mode. The scale ranged from *not at all* to *completely* willing. For the analyses, we combined the evaluation of the two scales using linear stretching, which resulted in a 11-point scale.

**Fig. 1:**
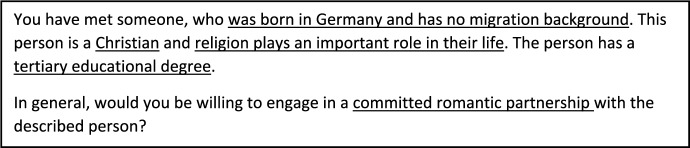
Example Vignette (English Translation). *Note*: Dimensions are underlined

The presented vignettes differed in four characteristics of the described partner as well as in the type of partnership respondents were asked to rate. Accordingly, the vignette encompassed five different dimensions, each with two or three levels (for an example of a vignette, see Fig. 1, and for details of the dimensions and levels, see Table S1 in the Supplementary Material), resulting in 96 unique vignettes in the full vignette universe. We employed a D-efficient design (Auspurg & Hinz, 2015), which allowed for all two-level interactions, to select 48 vignettes with unique level combinations, which were then allocated to twelve experimental groups. Each respondent was randomly assigned to one of those experimental groups, in which the order of the presented vignettes was randomly shuffled for each respondent. To make sure that the experimental groups were evenly distributed between respondents with and without migration background, we allocated the experimental groups separately for the two groups of respondents.

The factorial survey experiment offers several advantages over standard survey questions, such as allowing respondents to evaluate more complex and more realistic situations compared to isolated survey questions and reducing social desirability bias. Most importantly for our research question, the ability to simultaneously vary different characteristics of a potential partner allows for a better identification of the role of single factors that are often highly correlated with other characteristics in the real word. Accordingly, the FSE allows to better estimate the role of religion in partnership preferences compared to stylised question regarding the willingness to partner with someone of a specific denomination, where respondents might consider a “stereotypical” Muslim or Christian. Furthermore, the experimental design and the randomisation of vignette attributes to respondents reduce the risk of confounding and omitted variable bias by confounding factors. To make sure that the randomised assignment of respondents to the experimental vignette groups was effective, we compared our base model to a model controlling for different individual characteristics of respondents (gender, migration background, highest educational level, and partnership status). Since the results remained unchanged (see Table S2 in the Supplementary Material), we conclude that the experiment was successful and therefore do not control for individual characteristics in the subsequent analyses.

In the following analyses we focus on those three dimensions of the vignettes that allow us to test our hypotheses, i.e. information on the religious denomination of a potential partner—varying between being (1) Christian or (2) Muslim, the level of religiosity—varying between (1) religion not playing a big role or (2) religion playing an important role in the potential partner’s life, as well as the type of partnership—which either asked for the willingness to engage in (1) a casual partnership, (2) a committed partnership, or (3) a marriage with the described person. We introduced these different partnership types in the preface of the experiment and described a casual partnership to be less binding and oftentimes more short-term than a committed partnership. Furthermore, we instructed respondents who were in a romantic partnership at the time of the interview to answer the questions as if they were not in a partnership.

We additionally used information on respondents’ gender and denomination, making distinctions between individuals who identify as Muslim and those who identify as Christian. For this purpose, we combined respondents from different streams of Islam and Christianity. For respondents with missing information on the denomination, we imputed the respective information from wave 8. We excluded respondents that do not affiliate with any religious denomination, as our vignettes do not include fictious partners without religious denomination. Furthermore, we excluded respondents who affiliate with any denomination other than Islam or Christianity. To test our hypotheses on religious homophily in terms of denomination, we constructed a dummy variable ‘*same denomination’* with the value ‘1’ if the respondent has the same denomination as the potential partner described in the vignette and the value ‘0’ if otherwise.

After excluding respondents with missing information on their partnership status, gender or religious denomination, our analytical sample contains 9930 vignette evaluations by 2484 respondents. Descriptive statistics on the composition of the sample can be found in Table 1. In all following analyses, we employed linear regression analyses. As each respondent answered four vignettes, and hence the observations are not independent of each other, we estimated robust standard errors clustered by respondents. Additionally, we controlled for the order of the presented vignettes and the survey mode. It is important to note that we ran all models with the inclusion of all five vignette dimensions, but we present only the effects of those dimensions relevant to our hypotheses in the figures in the main text. The results of the full models can be found in the Supplementary Material.

**Table 1:** Description of variables

	Mean	Std. Dev	Min	Max
Vignette evaluation	6.13	3.37	1	11
	*N*		%	
*Religious denomination*				
Christian	2016		81.16	
Muslim	468		18.84	
*Gender*				
Men	949		38.20	
Women	1535		61.80	
*Survey mode*				
CAWI	2059		82.89	
PAPI	425		17.11	

*Notes*: CILS4EU-DE wave 9

## Results

### Religious Homophily by Denomination

To test our first two hypotheses—namely, that there is a higher preference for a partner of the same denomination (H1a) and that this effect is stronger for Muslims than Christians (H1b)—we estimate different models. First, we test the effect of belonging to the same denomination as the described vignette person on the willingness to engage in a partnership with this person. Second, we estimate a model that includes an interaction effect of respondents’ own religious denomination and their preference for a partner belonging to the same denomination, to determine whether this effect differs by religious denomination. Furthermore, we perform the same analyses for male and female respondents separately to see if these preferences differ by gender.

**Fig. 2:**
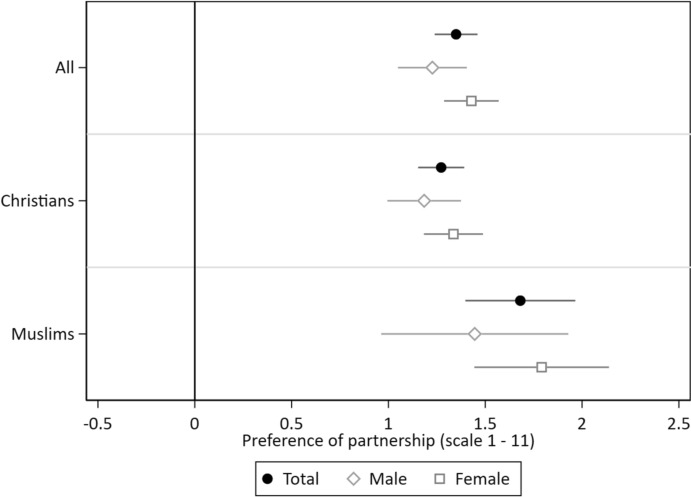
Preferences for a Partnership with a Person belonging to the same Denomination by Respondent’s Denomination and Gender. *Notes*: CILS4EU-DE wave 9. *N*(Total) = 9930 vignette evaluations by 2484 respondents; *N*(Male) = 3792 vignette evaluations by 949 respondents, *N*(Female) = 6138 vignette evaluations by 1535 respondents; the first row (All) shows beta-coefficients with 95% confidence intervals based on models reported in Table S3 in the Supplementary Material; the second and third rows show the conditional effects with 95 per cent confidence intervals based on Table S4 in the Supplementary Material

Figure 2 displays the results of these models. The first row of beta-coefficients in Fig. 2 (“All”) shows the effects on the willingness to engage in a romantic partnership with a partner belonging to the same denomination for all respondents without differentiating by the respondents’ gender (black circle) as well as the results of separate models for male (white diamond) and female (white square) respondents (see Table S3 in the Supplementary Material for the respective models). In line with hypothesis 1a, we find a statistically significant higher willingness to engage in a partnership if the potential partner belongs to the same denomination compared to a partner who belongs to a different denomination of 1.35 scale points on a 11-point scale.

The second (“Christians”) and third (“Muslims”) rows of Fig. 2 show the conditional effects of models in which we introduced an interaction term between respondents’ own denomination and whether the vignette person belongs to the same denomination as the respondent (see Table S4 in the Supplementary Material). In line with hypothesis 1b, the results show that preferences for a partner belonging to the aligning denomination are higher for Muslims than for Christians. Christian respondents report a 1.27 scale point higher willingness to partner with someone belonging to the same denomination than with someone belonging to a different denomination, whereas Muslim respondents report a 1.68 scale point higher willingness to partner with someone belonging to the same denomination. Accordingly, the difference in the preferences for a partner of the aligning denomination is 0.41 scale points stronger for Muslim respondents. This difference in effect size between Muslims and Christians is statistically significant on a 1%-level. Figure 2 also shows that the preference for partners of the aligning denomination for both Christian and Muslim women is slightly higher than for men. However, additional analyses testing a three-way interaction between respondents’ gender, respondents’ denomination as well as whether the vignette person belongs to the same denomination reveal that these gender differences are not statistically significant, providing no support for the assumption that the preference for a partner of the same denomination is stronger for (Muslim) women than men (see Table S5 in the Supplementary Material).

### The Role of Religiosity

**Fig. 3:**
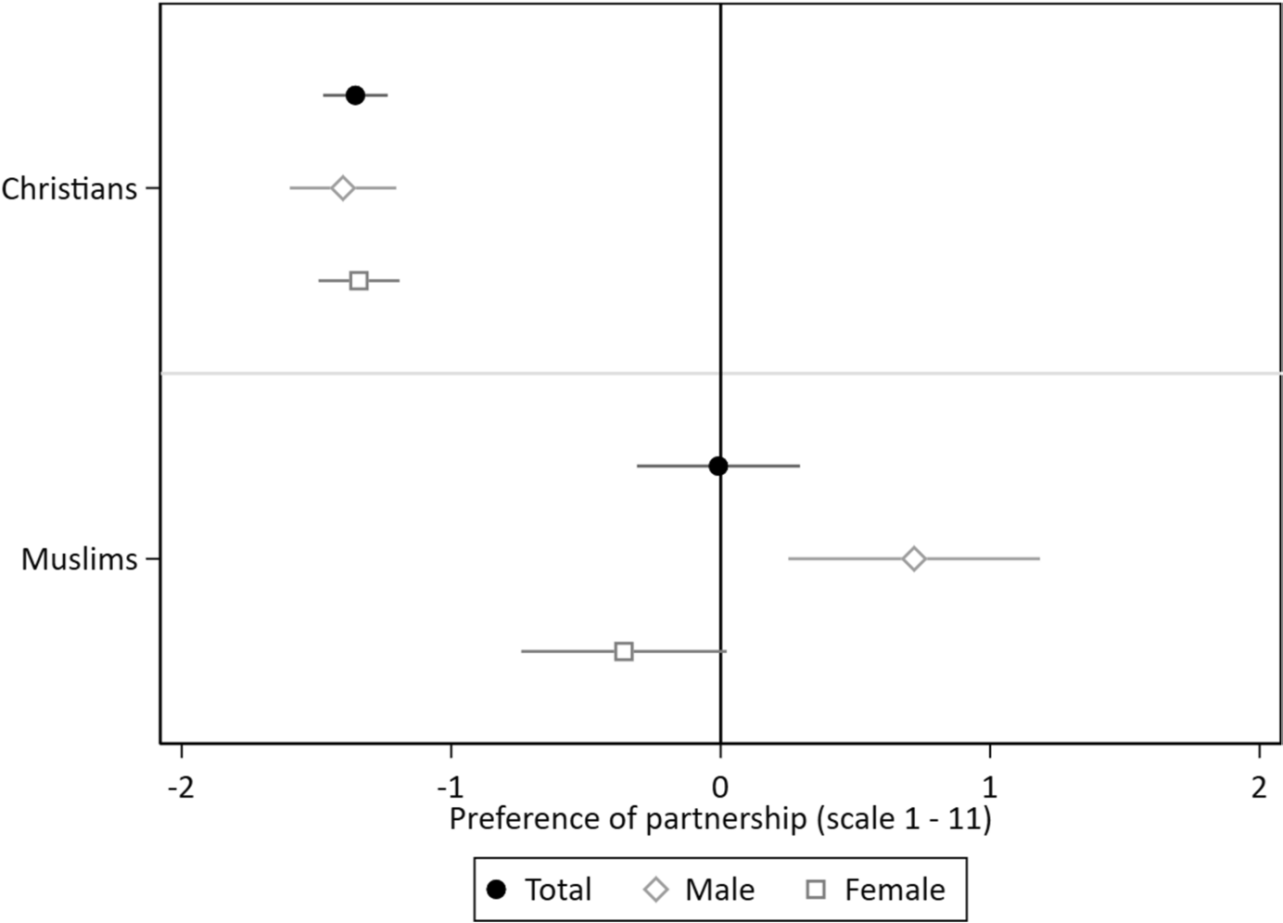
Preferences for a Partnership with a Religious Compared to a Non-Religious Vignette Person by Respondent’s Denomination and Gender. *Notes*: CILS4EU-DE wave 9. *N*(Total) = 9930 vignette evaluations by 2484 respondents; *N*(Male) = 3792 vignette evaluations by 949 respondents, *N*(Female) = 6138 vignette evaluations by 1535 respondents; conditional effect plots with 95 per cent confidence intervals. The estimates are reported in Table S6

We furthermore expect differences between Christian and Muslim respondents in their preference for religious versus non-religious partners (H2a). To test whether Muslims show a higher preference for religious over non-religious partners, while Christians prefer a non-religious partner over a religious partner, we estimate a model including an interaction term between respondents’ denomination and the religiosity of a potential partner. Again, we estimate the same model for male and female respondents separately in a second step. The results in Fig. 3 (see Table S6 in the Supplementary Material) show statistically significant differences for the effect of the religiosity of a potential partner between Christians and Muslims. Christian respondents show a clear disapproval of a religious partner with no differences between female and male respondents. Contrary to our expectation, Muslim respondents show on average no preference for a religious over a non-religious partner. However, this effect differs by the gender of the respondents. While male Muslim respondents display a slight preference for a more religious partner of 0.72 scale points, female Muslim respondents exhibit a slight preference for a non-religious partner of 0.36 scale points. Additional analyses show that this gender gap in preferences for a religious partner is statistically significant (see Table S7 in the Supplementary Material).

**Fig. 4:**
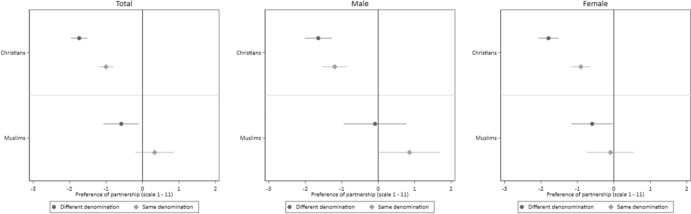
Gender Differences in Preferences for a Partnership with a Religious Compared to a Non-Religious Vignette Person by Denomination of Respondent and Vignette Person. *Notes*: CILS4EU-DE wave 9. *N*(Total) = 9930 vignette evaluations by 2484 respondents; *N*(Male) = 3792 vignette evaluations by 949 respondents, *N*(Female) = 6138 vignette evaluations by 1535 respondents; conditional effect plots with 95 per cent confidence intervals. The estimates are reported in Table S8

However, as previously discussed, the preference for the religiosity of a partner is expected to vary depending on the partner’s denomination, with a stronger preference anticipated for partners belonging to the aligning denomination (H2b). To test this assumption, we estimate three models (for all respondents, as well as for male and female respondents separately) in which we include an interaction between the religiosity of the vignette person, whether the vignette person belongs to the same denomination of the respondent, and the denomination of the respondents (see Fig. 4 and Table S8 in the Supplementary Material). Both Christian and Muslim respondents show a stronger preference for a religious partner when that partner belongs to the same denomination. Christian respondents overall prefer a non-religious partner over a religious partner, but this effect is statistically significantly smaller if this partner belonged to the same denomination. For Muslim respondents, the religiosity of a partner has no impact on their willingness to engage in a partnership if this partner is Muslim; however, they prefer a non-religious partner over a religious one if that partner is Christian.

The comparison of results for male and female respondents unveils noteworthy differences. Among male Christian respondents, the general disapproval of religious partners shows minimal variation and is not statistically significant between partners of the same denomination and those of a different denomination. In contrast, female Christian respondents exhibit a much stronger and statistically significantly higher disapproval of a religious partner if this partner belonged to a different denomination. For both male and female Muslim respondents, the effect of religiosity differs only slightly between partners of the same and those of a different denomination. While Muslim men show a preference for a religious partner if this partner belongs to the aligning denomination, Muslim women show a disapproval of a religious partner if this partner belongs to a different denomination.

Overall, our findings regarding the interplay of denomination and religiosity are partly in line with our expectations. Whereas all Christian respondents prefer a non-religious over a religious partner, this effect is smaller for Christian women if the partner belong to the same denomination. Contrary to our expectations, we do not find a generally higher preference for religious over non-religious partners among Muslims, and there are no significant differences based on partner’s denomination between male and female Muslim respondents.

### Religious Homophily by the Level of Partnership’s Commitment

**Fig. 5:**
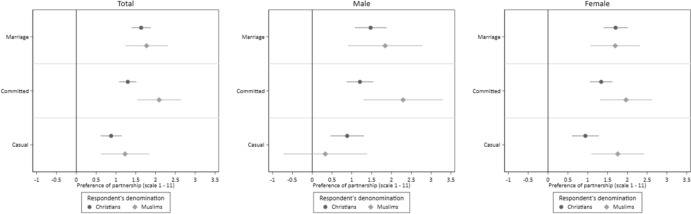
Preferences for a Partnership with a Person Belonging to the same Denomination by Type of Partnership and Respondent’s Denomination. *Notes*: CILS4EU-DE wave 9. *N*(Total) = 9930 vignette evaluations by 2484 respondents; *N*(Male) = 3792 vignette evaluations by 949 respondents, *N*(Female) = 6138 vignette evaluations by 1535 respondents; conditional effect plots with 95 per cent confidence intervals. The estimates are reported in Table S9

Finally, we take a closer look at the role of the level of commitment of a partnership. To test our assumption that religious homophily is stronger for partnerships with higher commitment, we estimate a model in which we included an interaction term between the denomination of the vignette person, the type of the partnership, and the denomination of the respondent. To detect potential gender differences, we estimate this model for all respondents, as well as for male and female respondents separately. Figure 5 illustrates the willingness of Christian and Muslim respondents to engage in various types of partnerships with individuals belonging to the same denomination.

Overall, we find that religious homophily differs by the type of partnership (H3a) and that this effect differs between Christian and Muslim respondents (H3b). The model for all respondents shows, that Christian respondents clearly prefer partners of the aligning denomination, but that this preference differed by partnership type. The preference for a partner of the same denomination is highest for marriage but is statistically significantly lower for dating. The preference for a committed partnership with a person of the same denomination is situated between these two effects.

For Muslim respondents, we find similarly high preferences for the partner of the same denomination across all partnership types, although this preference slightly diminishes for casual partnerships. When comparing Christian and Muslim respondents, we find a similarly strong preference for a partner of the same denomination for marriage. However, for committed partnerships, the preference for a partner belonging to the same denomination is stronger among Muslim respondents than among Christian respondents (statistically significant on 5% level), whereas the difference for casual partnerships is small.

The separate models for men and women indicate that this effect is primarily driven by male Muslim respondents, as we do not find a statistically significant higher preferences of dating someone belonging to the same denomination among them. In contrast, Muslim women exhibit consistently high preferences for partners of the aligning denomination regardless of the partnership type. For Christian respondents, we observe a similar pattern of preferences for partners belonging to the same denomination across both men and women. Overall, these finding are in line with the assumption that preferences for religiously homophile partnerships vary depending on the partnership type.

## Conclusions

In this study, we examined the role of religion for partnership preferences. Employing an innovative instrument—a factorial survey experiment—implemented in the 9th wave of the CILS4EU-DE survey, we (1) analysed whether young adults in Germany exhibited a preference for religiously homogeneous partnerships based on their own religious denomination, (2) examined whether this preference varied depending on the level of commitment in a partnership, and (3) explored gender differences in religious homophily. Overall, our results validated the significance of religion in shaping partnership choices among young Christian and Muslim adults in Germany. Furthermore, the study unveiled the heterogeneity in preferences for religious homogamy based on religious affiliation, gender, and the type of partnership.

Consistent with our expectations, we showed that both Christian and Muslim respondents tended to prefer partners from the same religious denomination. Notably, in line with our hypothesis, this preference was more pronounced among Muslim respondents compared to Christian respondents. Contrary to our expectations, these patterns were similar between female and male respondents.

Given the generally higher levels of religiosity among Muslims, our initial expectation was that Muslim respondents would exhibit a preference for more religious partners over less religious ones. However, this hypothesis could only be confirmed for Muslim men who showed a preference for a more religious partner, whereas we found no significant effect for the religiosity of a potential partner for Muslim women. When additionally taking into account the denomination of the potential partner, we find a preference for a religious partner if this partner belongs to the aligning denomination for Muslim men and a disapproval of a religious partner if this partner belongs to a different denomination for Muslim women. However, it is important to note that these differences in the acceptance of religious partners of different denominations between Muslim men and women are not statistically significant. The generally lower approval of religious partners among Muslim women compared to Muslim men could be explained by differences in the (anticipated) bargaining power in partnerships between men and women and that women perceive the religiosity of a partner as a greater threat to their autonomy. Although previous research has applied this argument mostly in the context of partnerships between majority group women compared to men who partner with a member of an outgroup (e.g. Dribe & Lundh, 2012), it is reasonable to assume that this argument also explains partnership preferences of young Muslim women who have been raised in the a society proposing more modern gender roles.

For Christian respondents, we observe a strong disapproval of the idea of having a partner who is highly religious for both men and women. While this disapproval of a religious partner does not differ by denomination of that partner for Christian men, Christian women show a lower disapproval of a religious partner, if the partner is Christian compared to Muslim. This finding could be by an interplay of the higher religiosity of Christian women compared to Christian men as shown in previous studies (e.g. Voas et al., 2013) and the perceived threat of the religiosity of a partner belonging to a different denomination for women as discussed above, resulting in a higher acceptance of religiosity of a Christian partner but not of a partner with a different denomination. However, future research is needed to dig deeper into the role of religiosity in partnership formation for men and women of different denominations.

Our analysis of the role of partnership commitment levels revealed a distinct pattern aligning with the winnowing thesis among Christian respondents. Their preference for partners sharing the same denomination was most pronounced for marriage, followed by committed partnerships, and least for casual relationships. Among Muslim respondents, a distinct preference for partners of the same religious denomination was evident across all types of partnerships, albeit to a lesser extent for casual relationships compared to marriage and committed partnerships. However, such a pattern was observed solely among male Muslims, with commitment levels not significantly impacting the preference for Muslim partners among female Muslims. These findings indicate some support for the winnowing thesis primarily among Muslim men, while such evidence is lacking for Muslim women.

These findings could potentially be explained by differing influences of third parties on Muslim and Christian young adults. Religious denominations may vary in the extent to which different types of partnerships are socially accepted, leading to varying degrees of social control over these partnerships. This social context may shape individuals’ preferences for partners of the same religious denomination, with Muslim respondents potentially facing stronger social pressure to seek partners within their own religious community compared to Christian respondents. Indeed, the observed differences between Muslims and other respondents regarding their attitudes towards various types of partnerships, as well as the gender disparities among Muslim respondents, could also be attributed to the influence of third parties such as parents and peers. Disentangling individual preferences from these external influences is challenging, even with the use of factorial survey experiments. This is because many values and preferences related to partnerships and partner characteristics are transmitted from parents to children through socialisation. As a result, individuals’ partnership preferences are often shaped not only by their personal beliefs and desires but also by societal norms and expectations instilled in them by their upbringing and social environment.

Due to the crucial role of individual preferences for partnership formation (Kalmijn, 1998), our study not only sheds light onto partnership preferences but might also contribute to the explanation of the still low share of interethnic unions in Western Europe. The factorial survey experiment employed in this study—compared to more general survey questions—is designed to disentangle highly correlated characteristics in the population, such as ethnicity and religion, thus allowing to study the importance of single characteristics for individual preferences in partnership formation. However, the realisation of these preferences in actual partnerships also depends on other factors, mainly the opportunity structure of the partner market (Blau, 1994). Future research should focus on the link between the preferences that are found in experimental settings, such as this study, and the realised interfaith (and other intergroup) partnerships. Special focus should thereby be placed on partnerships in early adulthood and more casual partnerships, since these partnerships and the experiences made during their formation and potential dissolution might be strong predictors of their future, more committed partnerships.

Another limitation of our study is that we were not able to analyse homophily preferences among young adults who are not affiliated with any religion and those who belong to other denominations than Christianity or Islam, as the design of the factorial survey experiment did not include potential partners outside these affiliations. Similarly, we could not differentiate between different branches of Christianity and Islam. Including potential partners without a religious affiliation would have resulted in implausible combinations of dimensions (e.g. a highly religious person without a religious denomination), necessitating their exclusion from the experiment and reducing our ability to study the effects of religious denomination and religiosity separately. Additionally, incorporating further levels of potential partners’ religious denomination (e.g. protestant Christians, Sunni Muslims, or Hindus and Jews) would have significantly expanded the vignette universe. This expansion would have required an increase in the number of vignettes for respondents, adding strain and potentially raising survey drop-out rates. Therefore, we opted against including additional religious groups. However, since interfaith marriages and the preferences for them may vary among religions not examined in this paper, future research should consider this diversity.

In conclusion, this study underscores the continuing importance of religious homophily in partnership preferences among young adults in Germany. The insights gained contribute to a deeper understanding of how religious identity and cultural background influence partnership choices in a multicultural society. Future research should further explore these dynamics by including diverse religious groups and examining how interfaith relationships evolve within the broader context of societal change. By addressing these aspects, scholars can enrich our understanding of social interactions in increasingly diverse communities and societies.

## Supplementary Information

Below is the link to the electronic supplementary material.

Supplementary file1 (PDF 393 kb)

## Data Availability

The datasets analysed in the current study are available in the GESIS Data Archive for the Social Sciences, under https://doi.org/10.4232/cils4eu-de.6656.7.0.0. The code for our analyses is available at 10.17605/OSF.IO/64AV3.

## Footnotes

1 By using terms like immigrants and their descendants or descendants of immigrants, we refer to individuals who have not immigrated themselves or were very young when they immigrated. This might include those who have been born in Western societies to immigrant parents or grandparents and those whose foreign origin lies even further in the past. In the case of Germany, the term descendants of immigrants mostly refers to those born in Germany to one or two immigrant parents or to those who have at least one grandparent who immigrated to Germany.

2 Respondents with migration background include those who themselves, one or both of their parents, any or all of their grandparents were not born in Germany (Dollmann et al., 2014). This means that the group of respondents without migration background (natives) includes only those who themselves, both parents, and all grandparents were born in Germany.
