# Supplementary material for: Partnership Preferences in Modern Migration Societies: Religious Homophily Among Young Muslims and Christians in Germany
Source: Eur J Popul. 2025 Jan 16;41(1):4. doi: 10.1007/s10680-024-09728-0 (PMC11739061; doi:10.1007/s10680-024-09728-0)
Supplement: Supplementary file 1 — Supplementary file1 (PDF 393 kb) [file 10680_2024_9728_MOESM1_ESM.pdf]

Partnership preferences in modern migration societies: Religious homophily among young Muslims and Christians in Germany

-Supplementary Material-

Table S1. Dimensions and Levels of the Vignettes (English Translation)

**Dimensions**	**Levels**
Type of partnership	1. Marriage<br>2. Committed romantic partnership<br>3. Casual romantic partnership
Characteristics of the described partner	
Origin	1. Born in Germany, without migration background (MB)<br>2. Born in Germany, with migration background<br>3. Syrian refugee<br>4. Afghan refugee
Religious denomination	1. Christian<br>2. Muslim
Religiosity	1. Religion does not play a big role in the person's life<br>2. Religion plays an important role in the person's life
Educational attainment	1. Never has been enrolled in tertiary education<br>2. Has a tertiary educational degree

Table S2. Results of the Factorial Survey Experiment with and without control variables

	W/o control variables	With control variables
VP belongs to the same denomination	1.35***	1.35***
	(0.06)	(0.06)
VP religious	-1.16***	-1.16***
	(0.06)	(0.06)
Type of partnership (Ref. Marriage)		
Committed	0.32***	0.31***
	(0.06)	(0.06)
Casual	0.16*	0.15*
	(0.08)	(0.07)
Origin of VP (Ref. German without MB)		
German with MB	0.11	0.11
	(0.07)	(0.07)
Syrian refugee	-1.02***	-1.02***
	(0.07)	(0.07)
Afghan refugee	-1.05***	-1.05***
	(0.07)	(0.07)
VP has tertiary education	0.55***	0.55***
	(0.05)	(0.05)
R. has migration background (Ref. None)		-0.83***
		(0.10)
R. is female (Ref. Male)		-0.92***
		(0.10)
R. educational level (ref. Tertiary degree)		
no degree		-0.54*
		(0.24)
lower secondary degree		-1.32***
		(0.27)
upper/intermediate secondary degree		-0.06
		(0.15)
vocational degree		-0.22
		(0.12)
R is in partnership		0.23*
		(0.11)
Constant	6.29***	7.36***
	(0.16)	(0.20)
N (vignette evaluations)	9,698	9,698
N (respondents)	2,426	2,426

Notes: MB = Migration background; VP = Vignette person; R = Respondent. Linear regression models controlling for vignette position and survey mode. Respondents with missing information on control variables are excluded in both models.
Source: CILS4EU-DE wave 9; Standard errors in parentheses. $^{*}p<.05$, $^{**}p<.01$, $^{***}p<.001$.

Table S3. Preferences for a Partnership with a Person belonging to the same Denomination by Respondent's Gender

	**All respondents**	**Male respondents**	**Female respondents**
VP belongs to the same denomination	1.35***	1.23***	1.43***
	(0.06)	(0.09)	(0.07)
VP religious	-1.14***	-1.13***	-1.15***
	(0.06)	(0.09)	(0.07)
Type of partnership (Ref. Marriage)			
Committed	0.32***	0.31**	0.30***
	(0.06)	(0.10)	(0.08)
Casual	0.16*	0.40***	-0.02
	(0.07)	(0.12)	(0.09)
Origin of VP (Ref. German without MB)			
German with MB	0.10	-0.01	0.17
	(0.07)	(0.10)	(0.09)
Syrian refugee	-1.01***	-0.89***	-1.10***
	(0.07)	(0.11)	(0.09)
Afghan refugee	-1.06***	-1.05***	-1.06***
	(0.07)	(0.11)	(0.08)
VP has tertiary education	0.55***	0.44***	0.62***
	(0.05)	(0.07)	(0.06)
Constant	6.28***	7.08***	5.67***
	(0.16)	(0.23)	(0.20)
N (vignette evaluations)	9,930	3,792	6,138
N (respondents)	2,484	949	1,535

Notes: MB = Migration background; VP = Vignette person; R = Respondent. Linear regression models controlling for vignette position and survey mode.
Source: CILS4EU-DE wave 9; Clustered standard errors in parentheses.
$^{*}p<.05$, $^{**}p<.01$, $^{***}p<.001$.

Table S4. Preferences for a Partnership with a Person belonging to the same Denomination by Respondent's Denomination and Gender.

	All respondents	Male respondents	Female respondents
Interaction effects			
VP belongs to the same denomination	1.27***	1.18***	1.34***
	(0.06)	(0.10)	(0.08)
Muslim respondent	-1.46***	-0.64*	-1.78***
	(0.16)	(0.28)	(0.19)
VP same denom # R Muslim	0.41**	0.26	0.46*
	(0.16)	(0.26)	(0.19)
VP religious	-1.14***	-1.13***	-1.14***
	(0.06)	(0.09)	(0.07)
Type of partnership (Ref. Marriage)			
Committed	0.32***	0.30**	0.30***
	(0.06)	(0.10)	(0.08)
Casual	0.16*	0.40***	-0.02
	(0.07)	(0.12)	(0.09)
Origin of VP (Ref. German without MB)			
German with MB	0.10	-0.00	0.16
	(0.07)	(0.10)	(0.09)
Syrian refugee	-1.01***	-0.89***	-1.10***
	(0.07)	(0.11)	(0.09)
Afghan refugee	-1.06***	-1.05***	-1.06***
	(0.07)	(0.11)	(0.08)
VP has tertiary education	0.55***	0.44***	0.61***
	(0.05)	(0.07)	(0.06)
Constant	6.67***	7.20***	6.25***
	(0.16)	(0.23)	(0.21)
N (vignette evaluations)	9,930	3,792	6,138
N (respondents)	2,484	949	1,535

Notes: MB = Migration background; VP = Vignette person; R = Respondent. Linear regression models controlling for vignette position and survey mode.
Source: CILS4EU-DE wave 9; Clustered standard errors in parentheses.
$^{*}p<.05$, $^{**}p<.01$, $^{***}p<.001$.

Table S5. Preferences for a Partnership with a Person belonging to the same Denomination in Interaction with Respondent's Denomination and Gender.

	Coef.	SE
Interaction effects		
VP belongs to the same denomination	1.18***	(0.10)
Muslim respondent	-0.63*	(0.28)
VP same denom # R Muslim	0.27	(0.26)
Female respondent	-0.77***	(0.13)
VP same denom # R female	0.15	(0.13)
R Muslim # R female	-1.17***	(0.33)
VP same denom # R Muslim # R female	0.19	(0.33)
VP religious	-1.13***	(0.06)
Type of partnership (Ref. Marriage)		
Committed	0.31***	(0.06)
Casual	0.15*	(0.07)
Origin of VP (Ref. German without MB)		
German with MB	0.10	(0.07)
Syrian refugee	-1.01***	(0.07)
Afghan refugee	-1.06***	(0.07)
VP has tertiary education	0.55***	(0.05)
Constant	7.11***	(0.17)
N (vignette evaluations)	9,930	
N (respondents)	2,484	

Notes: MB = Migration background; VP = Vignette person; R = Respondent. Linear regression models controlling for vignette position and survey mode.
Source: CILS4EU-DE wave 9; Clustered standard errors in parentheses.
$^{*}p<.05$, $^{**}p<.01$, $^{***}p<.001$.

Table S6. Preferences for a Partnership with a Religious compared to a Non-Religious Vignette Person by Respondent's Denomination and Gender.

	**All respondents**	**Male respondents**	**Female respondents**
Interaction effects			
VP strongly religious	-1.36***	-1.40***	-1.34***
	(0.06)	(0.10)	(0.08)
Muslim respondent	-1.93***	-1.56***	-2.04***
	(0.16)	(0.27)	(0.20)
VP religious # R Muslim	1.35***	2.12***	0.98***
	(0.17)	(0.26)	(0.21)
VP Muslim	-0.73***	-0.81***	-0.70***
	(0.06)	(0.09)	(0.08)
Type of partnership (Ref. Marriage)			
Committed	0.33***	0.30**	0.32***
	(0.06)	(0.10)	(0.08)
Casual	0.18*	0.38**	0.02
	(0.08)	(0.12)	(0.10)
Origin of VP (Ref. German without MB)			
German with MB	0.10	-0.01	0.17
	(0.07)	(0.10)	(0.09)
Syrian refugee	-1.01***	-0.85***	-1.11***
	(0.07)	(0.11)	(0.09)
Afghan refugee	-1.04***	-0.96***	-1.08***
	(0.07)	(0.11)	(0.09)
VP has tertiary education	0.57***	0.43***	0.65***
	(0.05)	(0.08)	(0.07)
Constant	7.74***	8.31***	7.32***
	(0.16)	(0.23)	(0.21)
N (vignette evaluations)	9,930	3,792	6,138
N (respondents)	2,484	949	1,535

Notes: MB = Migration background; VP = Vignette person; R = Respondent. Linear regression models controlling for vignette position and survey mode

Source: CILS4EU-DE wave 9; Standard errors in parentheses.

$^{*}p<.05$, $^{**}p<.01$, $^{***}p<.001$.

Table S7. Preferences for a Partnership with a Religious compared to a Non-Religious Vignette Person in Interaction Respondent's Denomination and Gender.

	**Coef.**	**SE**
Interaction effects		
VP strongly religious	-1.38***	(0.10)
Muslim respondent	-1.54***	(0.27)
VP strongly religious # R Muslim	2.10***	(0.26)
Female respondent	-0.72***	(0.13)
VP strongly religious # R female	0.05	(0.13)
R Muslim # R female	-0.51	(0.34)
VP strongly religious # R Muslim # R female	-1.12***	(0.33)
VP Muslim	-0.74***	(0.06)
Type of partnership (Ref. Marriage)		
Committed	0.32***	(0.06)
Casual	0.16*	(0.08)
Origin of VP (Ref. German without MB)		
German with MB	0.10	(0.07)
Syrian refugee	-1.00***	(0.07)
Afghan refugee	-1.04***	(0.07)
VP has tertiary education	0.56***	(0.05)
Constant	8.14***	(0.17)
N (vignette evaluations)	9,930	
N (respondents)	2,484	

Notes: MB = Migration background; VP = Vignette person; R = Respondent. Linear regression models controlling for vignette position and survey mode
Source: CILS4EU-DE wave 9; Standard errors in parentheses.
$^{*}p<.05$, $^{**}p<.01$, $^{***}p<.001$

Table S8. Gender Differences in Preferences for a Partnership with a Religious compared to a Non-Religious Vignette Person by Denomination of Respondent and Vignette Person.

	All respondents		Male respondents		Female respondents	
	**Coef.**	**SE**	**Coef.**	**SE**	**Coef.**	**SE**
Interaction effects						
VP religious	-1.74***	(0.11)	-1.64***	(0.19)	-1.79***	(0.14)
VP belongs to the same denomination	0.91***	(0.11)	0.97***	(0.17)	0.90***	(0.14)
VP religious # VP same denomination	0.74***	(0.19)	0.45	(0.30)	0.89***	(0.23)
Muslim respondent	-2.02***	(0.21)	-1.31***	(0.34)	-2.38***	(0.25)
VP religious # R Muslim	1.16***	(0.27)	1.56**	(0.48)	1.20***	(0.32)
VP same denom # R Muslim	0.26	(0.28)	-0.25	(0.47)	0.65	(0.34)
VP religious # VP same denom # R Muslim	0.18	(0.48)	0.50	(0.82)	-0.39	(0.57)
Vignette dimensions						
Type of partnership (Ref. Marriage)						
Committed	0.31***	(0.06)	0.29**	(0.10)	0.30***	(0.08)
Casual	0.16*	(0.07)	0.39***	(0.12)	-0.02	(0.09)
Origin of VP (Ref. German without MB)						
German with MB	0.10	(0.06)	-0.00	(0.10)	0.17	(0.09)
Syrian refugee	-1.02***	(0.07)	-0.88***	(0.11)	-1.11***	(0.08)
Afghan refugee	-1.06***	(0.06)	-1.02***	(0.11)	-1.07***	(0.08)
VP has tertiary education	0.55***	(0.05)	0.43***	(0.07)	0.62***	(0.06)
Constant	6.97***	(0.16)	7.44***	(0.24)	6.59***	(0.22)
N (vignette evaluations)	9,930		3,792		6,138	
N (respondents)	2,484		949		1,535	

Notes: MB = Migration background; VP = Vignette person; R = Respondent. Linear regression models controlling for vignette position and survey mode. *Source*: CILS4EU-DE wave 9; Standard errors in parentheses. $^{*}p<.05$, $^{**}p<.01$, $^{***}p<.001$.

Table S9. Preferences for a Partnership with a Person belonging to the same Denomination by Type of Partnership and Respondent's Denomination.

	All respondents		Male respondents		Female respondents	
	**Coef.**	**SE**	**Coef.**	**SE**	**Coef.**	**SE**
Interaction effects						
VP belongs to the same denomination	1.64***	(0.12)	1.47***	(0.21)	1.71***	(0.16)
Type of partnership (Ref. Marriage)						
Committed	0.51***	(0.11)	0.55**	(0.18)	0.46***	(0.13)
Casual	0.68***	(0.15)	0.82***	(0.24)	0.53**	(0.18)
VP same denomination # Type of partnership						
VP same denom # Committed romantic partnership	-0.34*	(0.17)	-0.27	(0.27)	-0.36	(0.21)
VP same denom # Casual romantic partnership	-0.76***	(0.21)	-0.59	(0.33)	-0.76**	(0.26)
Muslim Respondent	-1.02***	(0.22)	-0.20	(0.40)	-1.32***	(0.26)
VP same denom # R Muslim	0.14	(0.30)	0.37	(0.52)	-0.01	(0.35)
Type of partnership # Denomination of respondent						
Committed # R Muslim	-0.49	(0.27)	-1.04*	(0.49)	-0.21	(0.32)
Casual # R Muslim	-0.87**	(0.29)	-0.28	(0.51)	-1.23***	(0.33)
VP same denom # Type of partnership # Denomination of respondent						
VP same denom # Committed # R Muslim	0.66	(0.43)	0.72	(0.75)	0.64	(0.51)
VP same denom # Casual # R Muslim	0.22	(0.50)	-0.92	(0.85)	0.83	(0.57)
Vignette dimensions						
VP Religious	-1.14***	(0.06)	-1.13***	(0.09)	-1.15***	(0.07)
Origin of VP (Ref. German without MB)						
German with MB	0.10	(0.07)	0.00	(0.10)	0.17*	(0.09)
Syrian refugee	-1.02***	(0.07)	-0.88***	(0.11)	-1.11***	(0.08)
Afghan refugee	-1.06***	(0.07)	-1.04***	(0.11)	-1.07***	(0.08)
VP has tertiary education	0.54***	(0.05)	0.44***	(0.07)	0.60***	(0.06)

Constant	6.44***	(0.17)	6.98***	(0.26)	6.03***	(0.22)
N (vignette evaluations)	9,930		3,792		6,138	
N (respondents)	2,484		949		1,535	

Notes: MB = Migration background; VP = Vignette person; R = Respondent. Linear regression models controlling for vignette position and survey mode
Source: CILS4EU-DE wave 9; Standard errors in parentheses.
**p<.05,* ***p<.01,* ****p<.001*.
